# Self-monitoring of blood glucose during pregnancy: indications and limitations

**DOI:** 10.1186/1758-5996-4-54

**Published:** 2012-12-22

**Authors:** Carlos Antonio Negrato, Lenita Zajdenverg

**Affiliations:** 1 Gestational Diabetes Department of the Brazilian Diabetes Society, São Paulo, SP, Brazil; 2 Nutrology and Diabetes Unit, Universidade Federal do Rio de Janeiro, Rio de Janeiro, RJ, Brazil

**Keywords:** Self-monitoring of blood glucose, Diabetes mellitus, Pregnancy, Review

## Abstract

Self-monitoring of blood glucose (SMBG) is an important tool to treat diabetes during pregnancy. However, proper implementation of SMBG in pregnant women requires understanding of its applications and limitations. This article reviews issues related to the implementation, efficacy, and accuracy of SMBG and discusses factors that can confound results of SMBG during pregnancy.

## Introduction

Perinatal morbidity and mortality rates, often affected by maternal diabetes, have dramatically been reduced since the discovery of insulin and its therapeutic implementation. In addition to increased availability of insulin, many important technological advances have been developed over the preceding decades. These advances culminated in a larger array of diagnostic and therapeutic capabilities that contributed to improved outcomes in high-risk pregnancies.

The availability of glucose meters has represented an important positive impact in the treatment of pregnant women with any type of diabetes. Data frequently show patients who perform self-monitoring of blood glucose (SMBG) more strictly adhere to treatment programs due to increased comprehension regarding treatment and participation in the prescribed treatment regimen [1].

### Indications for self-monitoring of blood glucose during pregnancy complicated by diabetes

SMBG is an integral part of standard diabetes care [2]. It allows pregnant women and their healthcare providers to determine the most effective therapeutic modality (e.g. diet, physical activity, or insulin) to control glucose levels and reduce risks of diabetes-related complications. The number of daily tests required to adequately monitor blood glucose levels is specific to the patient and based on the recommendation of the practitioner [3]. Several characteristics, unique to each pregnant woman should be considered. For example, the type of treatment (diet and/or insulin), frequency and intensity of physical activity, and the risk of hypoglycemia. Additionally, SMBG makes patients feel more secure and comfortable using insulin since it allows early recognition of symptoms of hypoglycemia [4].

The indications for, and frequency of SMBG in pregnant women that are not under insulin treatment must be tailored to the individual. Patients must be trained to adjust the amount of food intake with the frequency, intensity, and timing of physical exercise. It is unclear whether SMBG alone leads to improved glycemic control in non-insulin treated subjects with type 2 diabetes. Additionally, there is no data in women with gestational diabetes mellitus (GDM) [5].

Measured glucose values need to be frequently checked to ensure both accuracy and the patient’s understanding of any alterations to prescribed treatment. For the vast majority of patients using insulin, SMBG is recommended three or more times per day. A more intensive SMBG regimen is indicated for women with pre-gestational type 1 or 2 diabetes. The aim is to reach adequate HbA1c levels safely without inducing hypoglycemia [3,4].

### When to monitor

Strict monitoring of postprandial glucose levels is paramount during pregnancy. Many studies have shown that postprandial hyperglycemia beyond the 16th week of pregnancy is the main predictor for fetal macrosomia [6,7]. Peak plasma glucose levels during pregnancy occur between 60 and 90 minutes after eating. It is recommended to perform SMBG one hour after food intake to evaluate potential adjustments in meal composition and/or in the prandial insulin dose. In special circumstances, like women with slowed gastric emptying, a high-fat meal, or women who use regular insulin for a prandial bolus, it might be more appropriate to perform SMBG two hours after meals instead of one. SMBG performed before eating is the most useful parameter to identify optimal basal insulin doses. Evaluating glycemic levels during the night is recommended to diagnose and prevent nocturnal hypoglycemia [8].

One randomized study [6] of 66 women with GDM observed better neonatal outcomes by aiming for 1-hour postprandial glucose levels less than 140 mg/dL as opposed to a preprandial target of 59 to 106 mg/dL. In another study, 61 women with type 1 diabetes were randomly assigned into two groups at 16 weeks gestation. Women either monitored blood glucose levels preprandially or postprandially. Postprandial capillary blood glucose monitoring significantly reduced the incidence of preeclampsia and neonatal triceps skinfold thickness compared to preprandial monitoring [9]. These studies have been criticized for not using comparable target blood glucose levels for pre- and post-prandial monitoring. Regardless, most specialists prefer postprandial testing at least partly, for the physiologic changes discussed earlier.

### Meter considerations

Diabetes education is paramount for proper use of glucose meters and interpretation of measurements. Commercially available glucose meters vary widely with respect to several characteristics, such as: blood sample volume, size, testing speed, memory capacity, type of technology applied, costs, and type of strips used [10]. All disposable blood glucose meters function by one of two mechanisms: reflectance (photometric) or electrochemical (amperometric). Meters using reflectance measure a change in color on the strip that occurs after an enzymatic reaction with whole blood. Meters using electrochemical mechanisms work with small volumes of blood to measure electrochemical potential based on glucose oxidation [11].

### Issues related to self-blood glucose monitoring during pregnancy complicated by diabetes

#### Accuracy

Accuracy of a blood glucose meter reflects how closely the measured value is to the actual value, while precision describes the reproducibility of serial measurements and is independent of the accuracy. For effective SMBG, it is imperative that meters be both accurate and precise in a series of values [12]. The best single measure of both accuracy and precision is the mean absolute relative error (MARE). MARE is calculated by taking the average for the set of individual absolute errors relative to a reference value. For example, measured values of both 90 and 110 mg/dl are in error by 10% given a reference value of 100 mg/dl [12].

The International Standards Organization (ISO), in conjunction with international regulatory authorities, healthcare providers, and device manufacturers, established standards to evaluate accuracy of blood glucose meters. ISO 15197 calls for a minimum accuracy where 95% of all measured values fall within 20% of reference values above 75 mg/dl, and within 15 mg of glucose values below 75 mg/dl [13]. According to the American Diabetes Association, 5% of the values for glucose performed with a given glucose meter should fall below 75 mg/dl for this meter be considered accurate. According to data currently available, the most accurate meter today has only 63% acceptable values in the 5% inaccuracy range [12]. Accordingly, it can be difficult for health care providers and patients to assess the accuracy of blood glucose monitoring systems. SMBG often presents significant errors that are generally poorly understood by patients and providers [14]. Healthcare providers can help patients to obtain better results by identifying the source of errors and methods for prevention and correction [12].

#### Inaccuracy of self-monitoring blood glucose during pregnancy complicated by diabetes

The inaccuracy of SBGM during pregnancy comes from four sources: strip factors, physical factors, patient factors, and pharmacological factors.

#### Strip factors

Small strip-to-strip variation may lead to inaccuracy in blood glucose readings. In some types of glucose strips, the size of individual reaction wells and changes in enzyme coverage on the strips may influence reading accuracy [12]. Reduction of the mediator can cause problems with the accuracy of electrochemical measurements. Normally, glucose is oxidized by glucose oxidase to form gluconic acid. The reduced form of glucose oxidase then interacts with water and oxygen to form hydrogen peroxide. On a strip, the first step is identical, but in the second step, the glucose oxidase pushes electrons to the oxidized mediator, causing its reduction, rather than oxygen plus water, forming hydrogen peroxide. The electrode oxidizes the mediator, generating the glucose signal [12].

The oxidized mediator is somewhat unstable and susceptible to reduction, particularly at high temperatures, which produces erroneously high blood glucose measurements [15]. Blood glucose strips require complex biochemical reactions to function properly, and have finite lifetimes, usually about 2 years under ideal storage conditions. Storing strips at high temperature or humidity, or in an open vial (allowing the humidity to get to the strips) can shorten the lifetime of the strips. Different brands of glucose strips fail differently. When a failure occurs, some brands underestimate the glucose values, whereas others overestimate. In both cases, the error can be large, and usually meters are unable to detect failed strips [12].

#### Physical factors

The most common physical factors that influence the accuracy of blood glucose strips are altitude and temperature [12].

Glucose oxidase biosensor strips are sensitive to oxygen concentrations. The mediator and oxygen can both compete as oxidants of the reduced form of glucose oxidase. Since the electrode will only detect the mediator, excess oxygen robs electrons from the active mediator leading to underestimated glucose values. Conversely, at lower oxygen concentrations, meters will overestimate blood glucose levels. Consequently, glucose oxidase biosensor strips are generally calibrated with capillary blood and are most accurate when used with capillary blood. Strips that use glucose dehydrogenase as the enzyme are less affected by oxygen, and therefore are less affected by altitude [12].

The influence of temperature is less predictable. Errors presented at extreme temperatures are brand-specific and not technology dependent. The errors may vary from 5-7%, but can be either positive or negative [12]. Low temperatures decrease circulation to the skin. This does not greatly influence glucose measured from a fingertip since the arteriovenous shunts of the fingers stay open. However, blood flow to the skin of the forearm is dramatically decreased. Alternative site testing, which normally has a lag of 15–30 min, can have a lag of up to an hour when the arm is exposed to a very low temperature [16].

#### Patient factors

The ability of a patient to use her meter properly can greatly influence the accuracy of a blood glucose measurement. Most blood glucose meters need to be coded. Coding determines the relationship between the electrical signal produced by the strip and the reported blood glucose. Improper coding is one of the most common mistakes made by patients and the effect of miscoding is not uniform across all blood glucose ranges [12]. Meters that do not require coding are more readily available and are more accurate when used by patients.

Hematocrit abnormalities can also affect the results obtained from SMBG. Hematocrit less than 30% may overestimate blood glucose, while hematocrit greater than 55% may underestimate measurements [17]. All meters use whole blood concentration from capillaries. When venous blood collected in laboratories, blood glucose is measured with respect to plasma and not whole blood. The density of erythrocytes causes glucose content to be 10% to 15% lower in whole blood than in plasma. However, most new meters convert whole-blood results into plasma-calibrated results [10].

Plasma glucose levels in individuals with normal hematocrit is 11% higher than in whole blood (venous or capillary). The International Federation of Clinical Chemistry and Laboratory Medicine (IFCC) recommends expressing plasma glucose values rather than whole blood values. Expression of plasma glucose values facilitates comparison with measurements obtained by laboratories. Plasma values can be obtained by multiplying whole blood glucose concentration by 1.1. Many glucose meters already convert blood values to plasma values. The glucose meter manufacturer should expressly state whether a meter automatically converts the whole blood measurement to a plasma measurement.

SMBG devices are frequently updating styles, models, and technologies. Systems are becoming more accurate and easier for patients to use appropriately. Hand washing has always been problematic, but because many newer meters use microsamples, small amounts of contaminant can greatly alter results [12]. Some substances that occur naturally in the body can affect the accuracy of electrochemical blood glucose meters. These include triglycerides, oxygen, and uric acid. High levels of triglycerides cause underestimation of blood glucose by displacing glucose in the capillary volume. Many pregnant women present with high triglycerides levels, especially in the third trimester, and this could lead to inaccuracy in their SMBG values. Oxygen competes with the mediator to take electrons from reduced glucose oxidase and high oxygen values, such as those found in arterial blood or in patients utilizing oxygen, will underestimate glucose values [12,18]. Low oxygen levels, in venous blood or patients with severe chronic obstructive pulmonary disease may overestimate glucose values. Extremely high levels of uric acid can give incorrectly high values. Uric acid is only problematic in patients with uric acid levels that are sufficient to induce severe gout [12,19].

Glucose dehydrogenase is a less specific enzyme, so some naturally occurring sugars can compete with glucose. Galactose, xylose, and maltose compete with glucose for glucose dehydrogenase binding sites and high levels of these other sugars can cause overestimations of glucose levels. However, glucose dehydrogenase sensors are less sensitive to variations in oxygen concentration [20].

#### Pharmacologic factors

Many medications can affect the readings from SMBG. With electrochemical glucose oxidase systems, acetaminophen, L-dopa, tolazamide, and ascorbic acid interact with the electrode [19]. Fortunately, the error from these medications is usually small. With glucose dehydrogenase, many other sugars can interfere. Maltose and xylose can have a small effect, but icodextrin can have a tremendous effect [21]. Icodextrin is used in some peritoneal dialysis fluids and can increase glucose value reported by the meter by more than 100 mg/dl. Meter manufacturers generally do not disclose what substances interfere with their specific product. However, doing so when possible would improve accuracy of SMBG measurements by patients and ultimate therapeutic efficacy.

#### Barriers to self-monitoring of blood glucose during pregnancy

The first step towards a successful SMBG during pregnancy is patient education and an understanding of the importance of SMBG to reducing complications during and after pregnancy. The patient must be properly educated on all aspects of meter use. It is important she be aware of how to properly code her meter, wash her hands prior to the test, and to apply the correct amount of blood to the test strip. It is also critical to educate patients on how glucose from food can affect the test results, to use test strips before the expiration date and not longer than 90 days after the vial was opened. Lastly, it is crucial to educate patients on proper storage of strips and disposal of strips if they are subjected to extreme humidity or temperature. Other common barriers to SMBG include costs of the meters and strips, lower socio-economic status, fewer HbA1c tests, obesity and other comorbidities, poor glycemic control, stigmas of testing in public places, pain, and inconvenience [4,22].

During pregnancy, relatively small changes in glucose values may be clinically important. In clinical practice, it is important to know how closely the glucose meter readings can reflect the patient’s real plasma glucose level. Perera et al. recently showed differences of up to 15% between SMBG and plasma glucose values. Variations of up to 15 mg/dl were found in pregnant women with diabetes [23]. On the other hand, Kong et al. found correlation between the SMBG and plasma glucose levels in 90% of the measurements performed in women with GDM that reached normoglycemia after dietary treatment exclusively [24].

## Conclusions

Treating hyperglycemia during pregnancy reduces adverse pregnancy outcomes. The first step towards a tight glucose control in pregnancy is patient adherence to SMBG. There are several barriers and sources of errors associated with SMBG. Pregnancy is a unique short period of time when physicians, diabetes educators, and healthcare professionals in general have the chance to provide information and education about diabetes and SMBG. Specifically, it is an opportunity to educate women on error prevention and correct interpretation of results. Additionally, they can provide assistance to overcome barriers associated with proper diabetes care and provide long-lasting benefits to the mother and fetus.

## Competing interests

Authors do not have any competing interests (financial, political, personal, religious, ideological, academic, intellectual, commercial or any other) to declare in relation to this manuscript.

## Authors’ contributions

CAN and LZ participated in article design and coordination and helped to draft the manuscript. All authors read and approved the final manuscript.
